# Generalizability of a biopsy-trained artificial intelligence algorithm to TURP and HoLEP specimens for detection of incidental prostate adenocarcinoma

**DOI:** 10.1186/s13000-026-01846-z

**Published:** 2026-08-26

**Authors:** Neşe Yeldir, Burak Uzel, Elif Nur Tekin, Gizem Solmaz Yılmaz, Eren Tekin, Sercan Çayır, İbrahim Serdar Coşkun, Bahar Müezzinoğlu

**Affiliations:** 1 Pathology Department, Istanbul Medipol University, Istanbul, Türkiye; 2 Virasoft, 1501 Broadway, 12th Floor, New York, NY 10036 USA; 3 Faculty of Medicine, Istanbul Medipol University, Istanbul, Türkiye

**Keywords:** Prostatic adenocarcinoma, Gleason score, Grade group, Artificial intelligence based algorithm, Digital pathology

## Abstract

**Background:**

Incidental prostatic adenocarcinoma is frequently identified in transurethral resection of the prostate (TURP) and holmium laser enucleation of the prostate (HoLEP) specimens obtained for the management of benign prostatic hyperplasia. Artificial intelligence (AI)-based algorithms have demonstrated promising performance in prostate biopsy assessment; however, their applicability to TURP and HoLEP materials remains insufficiently studied. We evaluated the standalone performance of an AI algorithm trained on prostate needle biopsies for detecting incidental adenocarcinoma in TURP and HoLEP specimens.

**Methods:**

A total of 3,241 whole-slide images from 368 consecutive patients (247 HoLEP and 121 TURP cases) received between May 2023 and December 2025 were retrospectively analyzed. All slides were scanned at ×20 magnification using a Hamamatsu NanoZoomer S360 scanner and processed using a commercially available AI algorithm. The algorithm generated heatmap masks highlighting regions suspicious for malignancy. Conventional histopathological diagnosis served as the reference standard. Sensitivity, specificity, positive predictive value (PPV), negative predictive value (NPV), accuracy, and area under the receiver operating characteristic curve (AUC-ROC) were calculated at the case level. The Cochran–Armitage trend test was used to assess the relationship between the number of AI-flagged slides and malignancy rates.

**Results:**

Thirty-five cases contained adenocarcinoma, of which 33 were correctly identified by the algorithm, yielding an overall sensitivity of 94.3%. Specificity was 76.0%, PPV 29.2%, NPV 99.2%, and accuracy 77.7%. Sensitivity was 92.3% for HoLEP specimens and 100% for TURP specimens. The case-level AUC-ROC was 0.89. Malignancy rates increased significantly with the number of AI-flagged suspicious slides (0.8%, 13.6%, 18.8%, and 44.4% for 0, 1, 2, and ≥ 3 suspicious slides, respectively; *p* < 0.001). The two false-negative cases were low-volume Grade Group 1 tumors.

**Conclusions:**

A biopsy-trained AI algorithm demonstrated high sensitivity and excellent negative predictive value for detecting incidental prostatic adenocarcinoma in TURP and HoLEP specimens. These findings support its potential role as a screening and workflow-assistance tool in routine uropathology practice.

## Introduction

Prostate cancer is one of the most frequently diagnosed malignancies among men worldwide and remains a major cause of cancer-related mortality [1]. Although needle biopsy represents the standard diagnostic approach, incidental prostatic adenocarcinoma continues to be identified in transurethral resection of the prostate (TURP) and holmium laser enucleation of the prostate (HoLEP) specimens obtained for benign prostatic hyperplasia. Despite declining incidence in the prostate-specific antigen (PSA) era, these incidental tumors retain clinical significance, particularly in patients without prior biopsy or radiologic suspicion [2, 3].

Histopathologic evaluation of TURP and HoLEP materials presents distinct practical and diagnostic challenges. These specimens are typically composed of numerous fragmented tissue chips distributed across multiple slides, resulting in substantial microscopic workload [4]. In addition, HoLEP specimens frequently demonstrate laser-related thermal artifacts, stromal fragmentation, tissue distortion, and discontinuous glandular architecture, whereas TURP specimens generally show relatively better-preserved morphology. Malignant foci may be sparse, focal, and morphologically subtle, increasing the risk of oversight during routine slide review [5].

Recent advances in digital pathology and whole-slide imaging (WSI) have enabled the integration of AI-based diagnostic support systems into routine pathology workflows [6, 7]. Currently, AI-based algorithms are increasingly used for the detection of prostatic adenocarcinoma, particularly in TRUS needle biopsy specimens [8, 9]. Multiple deep learning models trained on biopsy materials have demonstrated high diagnostic accuracy, sensitivity, and reproducibility in identifying malignant glands while potentially reducing pathologist workload and false-negative diagnoses [10, 11]. However, these algorithms have largely been developed and validated using relatively well-preserved core biopsy tissues. Unlike core biopsies, TURP and HoLEP materials exhibit substantial architectural distortion, tissue fragmentation, cautery-related artifacts, and scattered glandular distribution, all of which may adversely affect algorithmic performance [12]. Therefore, the generalizability of biopsy-trained AI algorithms to endoscopic prostate resection specimens remains insufficiently explored.

In this study, we evaluated the standalone diagnostic performance of a commercially available AI-based prostate cancer detection algorithm, originally trained on needle biopsy specimens, in a large real-world cohort of TURP and HoLEP whole-slide images. In addition to conventional diagnostic accuracy metrics, we investigated the relationship between the number of algorithm-flagged suspicious slides and the likelihood of malignancy, with the aim of assessing the potential utility of AI-assisted triage in routine uropathology practice.

## Methods

### Study cohort

This retrospective study included consecutive TURP and HoLEP specimens received at our institution between May 2023 and December 2025. A total of 368 patients were included, comprising 247 HoLEP cases and 121 TURP cases. Overall, 3241 hematoxylin and eosin (H&E)-stained WSIs were analyzed, including 2466 HoLEP slides and 775 TURP slides.

The majority of procedures were performed for benign prostatic hyperplasia; however, five patients had a prior diagnosis or strong clinical suspicion of prostatic adenocarcinoma at the time of surgery. Cases lacking complete pathology records or adequate digital slide quality were excluded from the study.

Histopathologic diagnosis established by conventional light microscopy served as the reference standard for adenocarcinoma detection.

### Digital slide scanning and AI-based analysis

All H&E-stained slides were digitized using a Hamamatsu NanoZoomer S360 scanner (Hamamatsu Photonics, Hamamatsu, Japan) at ×20 magnification and stored in NDPI format. Whole-slide images were analyzed using Virasoft’s commercially available AI-based prostate cancer detection algorithm originally developed and trained for needle biopsy specimens. The algorithm evaluated the entire WSI and generated heatmap overlays highlighting regions considered suspicious for prostatic adenocarcinoma.

The AI system was used by technicians and gave its output in a standalone manner. Pathologists were not assisted by algorithmic outputs during the original diagnostic process, and routine diagnoses were established independently using conventional light microscopy. Cases were considered AI-positive when at least one slide within the case was flagged as suspicious by the algorithm.

The AI system was developed using a weakly supervised, multiple instance learning framework designed for whole-slide image analysis. In this approach, each slide was represented as a bag of image tiles extracted from tissue-containing regions, while the diagnostic label was assigned at the slide-level. Tile-level annotations were not required during training. A pretrained histopathology image encoder was used to extract compact feature representations from tissue tiles, and these representations were subsequently aggregated by a slide-level learning module to generate a final prediction for adenocarcinoma presence.

The model was trained using a 5-fold cross-validation strategy. The cross-validation splits were generated in a stratified manner to preserve the distribution of positive and negative cases across folds as much as possible. To prevent data leakage, all slides belonging to the same case were assigned exclusively to a single split; therefore, no slides from the same case were present simultaneously in the training and validation sets.

During model development, several regularization and optimization strategies were explored to reduce overfitting and improve generalizability. These included the use of dropout within the classification module, weight decay during optimization, and learning rate scheduling throughout training. Hyperparameters related to these components were tuned during the training process.

### Light microscopy evaluation

All slides were routinely reviewed by experienced pathologists using conventional light microscopy. Diagnoses of prostatic adenocarcinoma, Gleason score, grade group, and estimated tumor volume were recorded according to routine pathology practice.

Slides identified as suspicious by the AI algorithm, as well as false-negative cases missed by the algorithm, underwent additional review to assess histopathologic correlates associated with algorithmic performance, including tumor volume, architectural distortion, thermal artifact, and glandular fragmentation.

### Statistical analysis

Diagnostic performance metrics including sensitivity, specificity, positive predictive value (PPV), negative predictive value (NPV), and overall accuracy were calculated separately for HoLEP and TURP specimens and for the combined cohort.

Receiver operating characteristic (ROC) analysis was performed at the case level based on overall WSI assessment, and the area under the curve (AUC-ROC) was calculated to evaluate discriminative performance.

To investigate the relationship between the number of AI-flagged suspicious slides and malignancy detection rates, the Cochran–Armitage trend test was applied. Statistical significance was defined as a two-sided p value < 0.05. All statistical analyses were performed using SPSS version 27.

## Results

A total of 368 patients and 3241 WSIs were included in the study, comprising 247 HoLEP cases (2466 slides) and 121 TURP cases (775 slides). Histopathologic examination identified incidental prostatic adenocarcinoma in 35 cases overall, including 26 HoLEP and 9 TURP specimens.

### Algorithm performance in HoLEP specimens

**Fig. 1:**
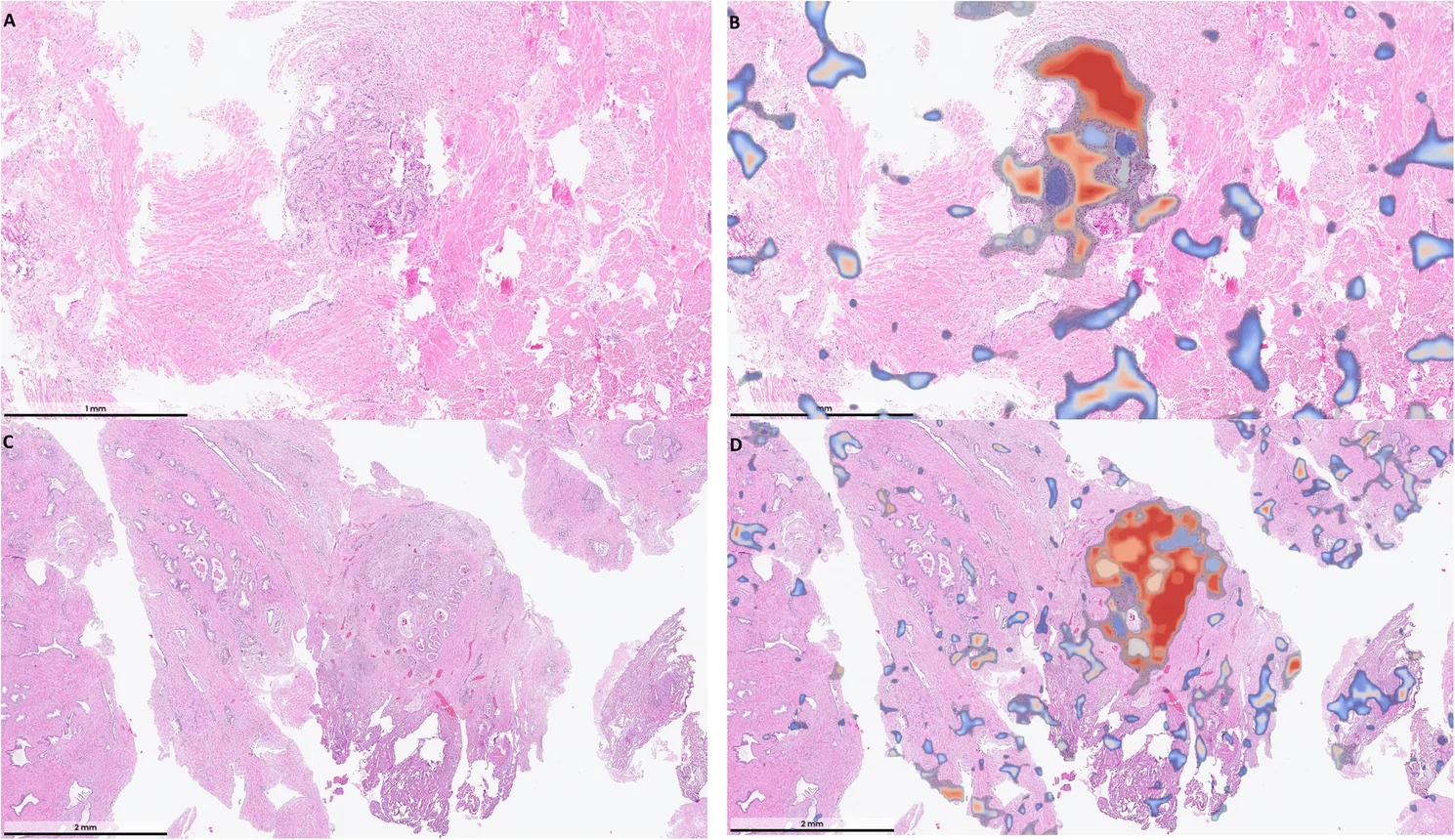
Representative HoLEP whole-slide image (WSI) examples analyzed by the AI-based prostate adenocarcinoma detection system. **A**, **B** Benign HoLEP case falsely classified as suspicious by the algorithm, with an overall malignancy probability score of 61%. **A** Histomorphologic appearance of the suspicious region showing benign glandular structures with artifact-related architectural distortion. **B** Corresponding AI-generated heatmap overlay demonstrating red-highlighted suspicious areas identified by the algorithm. **C**, **D** HoLEP case diagnosed as prostatic adenocarcinoma (Gleason score 3 + 3=6, Grade Group 1), for which the algorithm generated a malignancy probability score of 78%. **C** Histomorphologic appearance of the adenocarcinoma focus. **D** Corresponding AI-generated heatmap overlay highlighting the malignant region in red

**Fig. 2:**
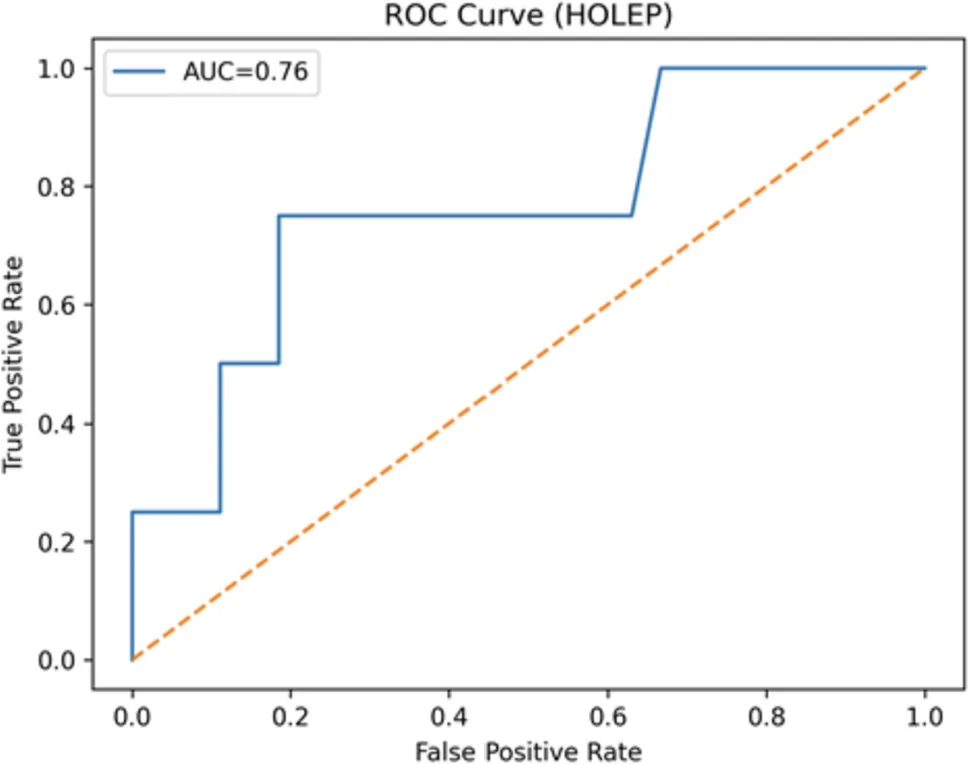
ROC curve demonstrating the diagnostic performance of the artificial intelligence-based algorithm for detection of incidental prostatic adenocarcinoma in HoLEP specimens. The algorithm achieved an AUC of 0.76, indicating moderate discriminative ability for distinguishing malignant from benign cases

Among the 247 HoLEP cases, the AI algorithm correctly identified 24 of 26 adenocarcinoma cases, yielding a sensitivity of 92.3%. Fifty-five benign cases were falsely classified as suspicious, corresponding to a specificity of 75.1%. The PPV and NPV were 30.4% and 98.8%, respectively. Overall diagnostic accuracy for HoLEP specimens was 76.9% (Figs.1 and 2).

The two false-negative HoLEP cases demonstrated low-volume Gleason score 6 (Grade Group 1) adenocarcinoma involving approximately 2% and 5% of the tissue, respectively. Both cases exhibited limited glandular atypia and minimal architectural distortion. Corresponding PSA levels were 3.1 ng/mL and 4.2 ng/mL.

### Algorithm performance in TURP specimens

**Fig. 3:**
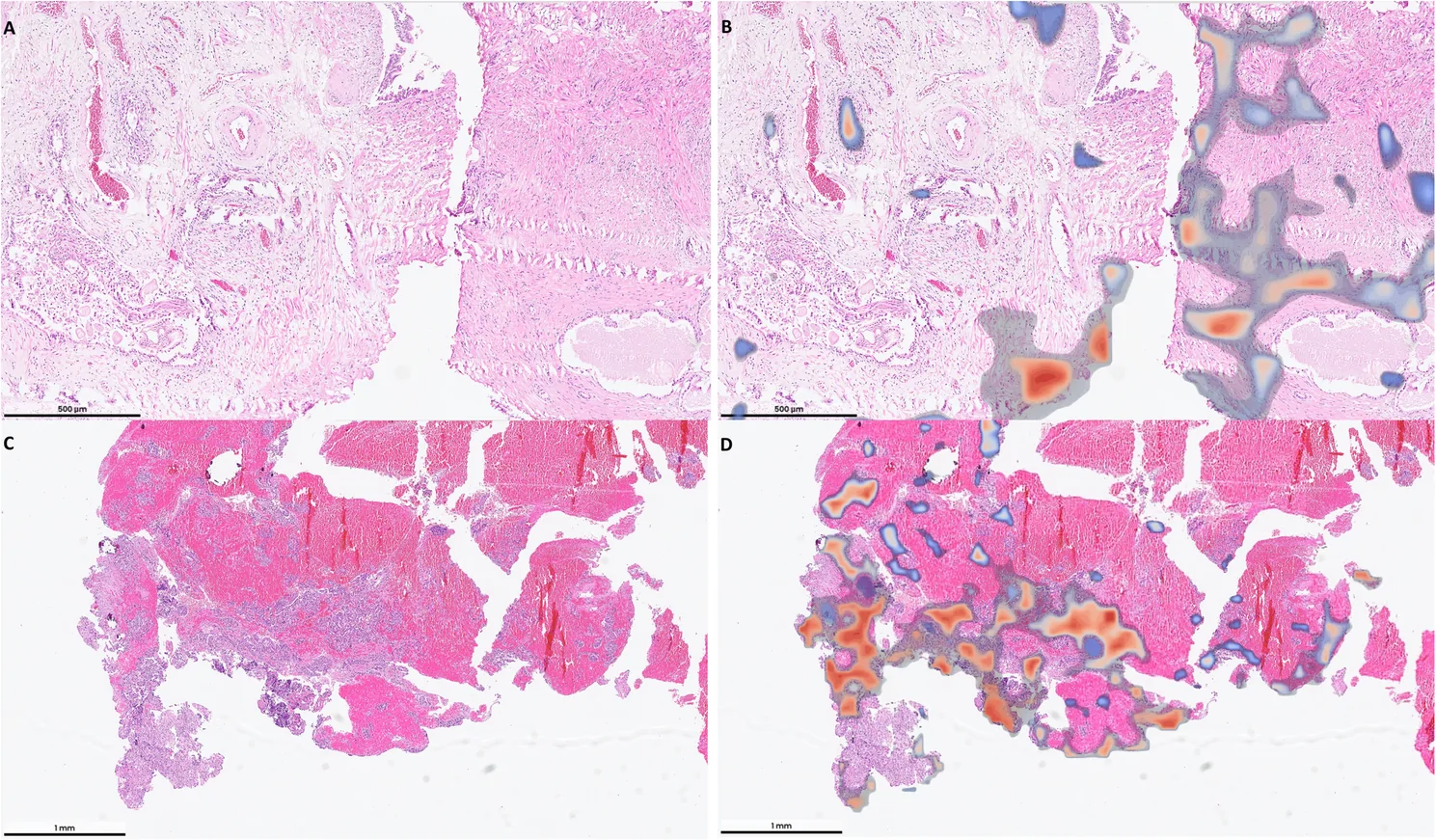
Representative TURP whole-slide image (WSI) examples analyzed by the AI-based prostate adenocarcinoma detection system. **A**, **B** Benign TURP case falsely classified as suspicious by the algorithm, with an overall malignancy probability score of 53%. **A** Histomorphologic appearance of the suspicious region showing benign glandular structures. **B** Corresponding AI-generated heatmap overlay demonstrating red-highlighted suspicious areas identified by the algorithm. **C**, **D** TURP case diagnosed as prostatic adenocarcinoma (Gleason score 4 + 5=9, Grade Group 5), for which the algorithm generated a malignancy probability score of 97%. **C** Histomorphologic appearance of the adenocarcinoma focus. **D** Corresponding AI-generated heatmap overlay highlighting the malignant region in red

**Fig. 4:**
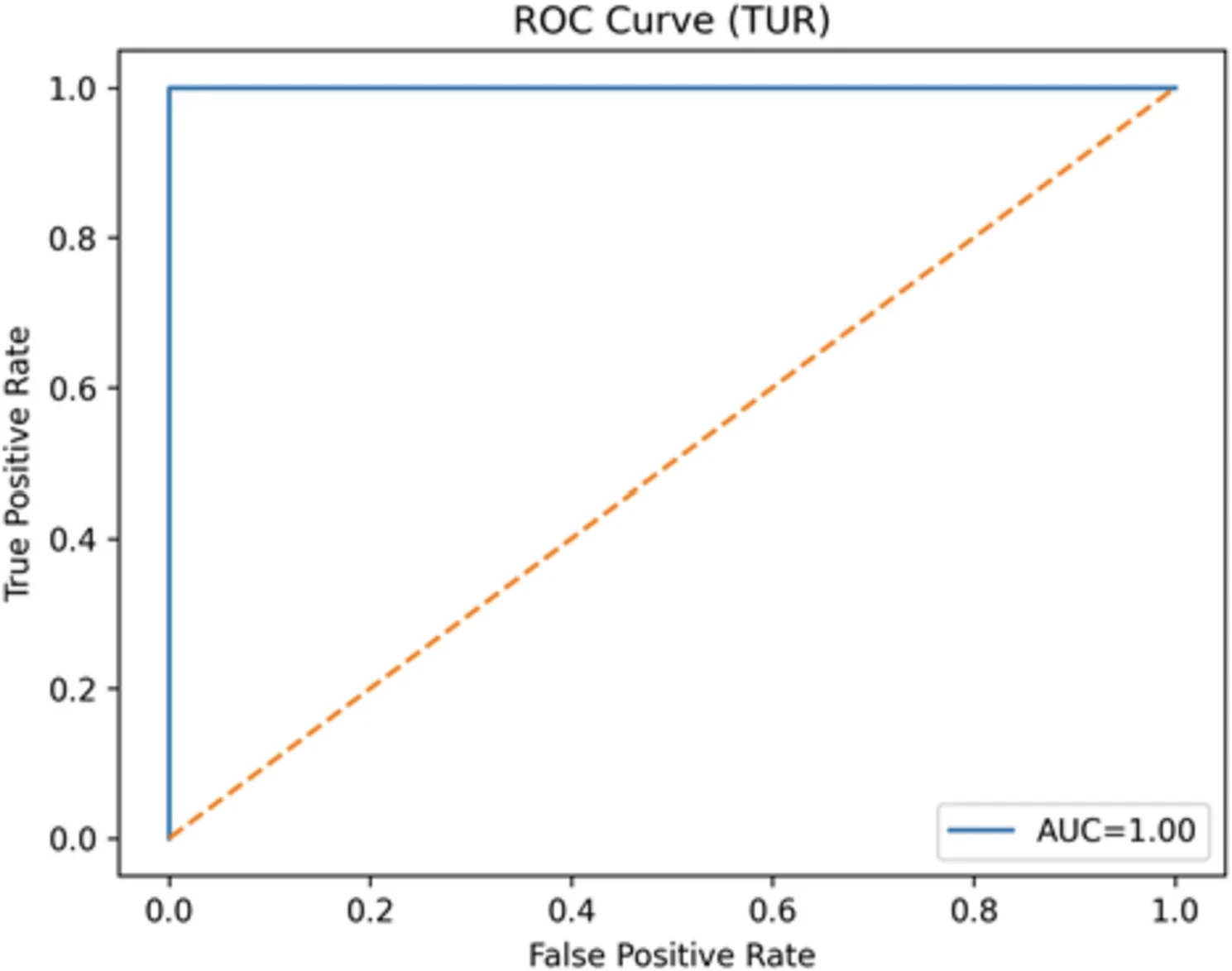
ROC curve demonstrating the diagnostic performance of the artificial intelligence-based algorithm for detection of incidental prostatic adenocarcinoma in TURP specimens. The algorithm achieved an AUC of 1.00, indicating excellent discriminative performance in distinguishing malignant from benign cases

Among the 121 TURP cases, all 9 adenocarcinoma cases were correctly identified by the algorithm, resulting in a sensitivity of 100%. Twenty-five benign cases were falsely flagged as suspicious, producing a specificity of 77.7%. The PPV and NPV were 26.5% and 100%, respectively. Overall diagnostic accuracy in TURP specimens was 79.3% (Figs. 3 and 4).

Compared with HoLEP materials, TURP specimens generally demonstrated better-preserved glandular architecture and fewer thermal artifacts, which may have contributed to slightly improved algorithmic performance.

### Overall diagnostic performance

When both specimen types were analyzed together, the AI algorithm achieved an overall sensitivity of 94.3%, specificity of 76.0%, PPV of 29.2%, NPV of 99.2%, and diagnostic accuracy of 77.7%.

ROC analysis performed at the case level demonstrated an AUC-ROC of 0.89, indicating strong discriminative ability for identifying incidental adenocarcinoma in TURP and HoLEP specimens.

### Relationship between number of suspicious slides and malignancy rate

**Fig. 5:**
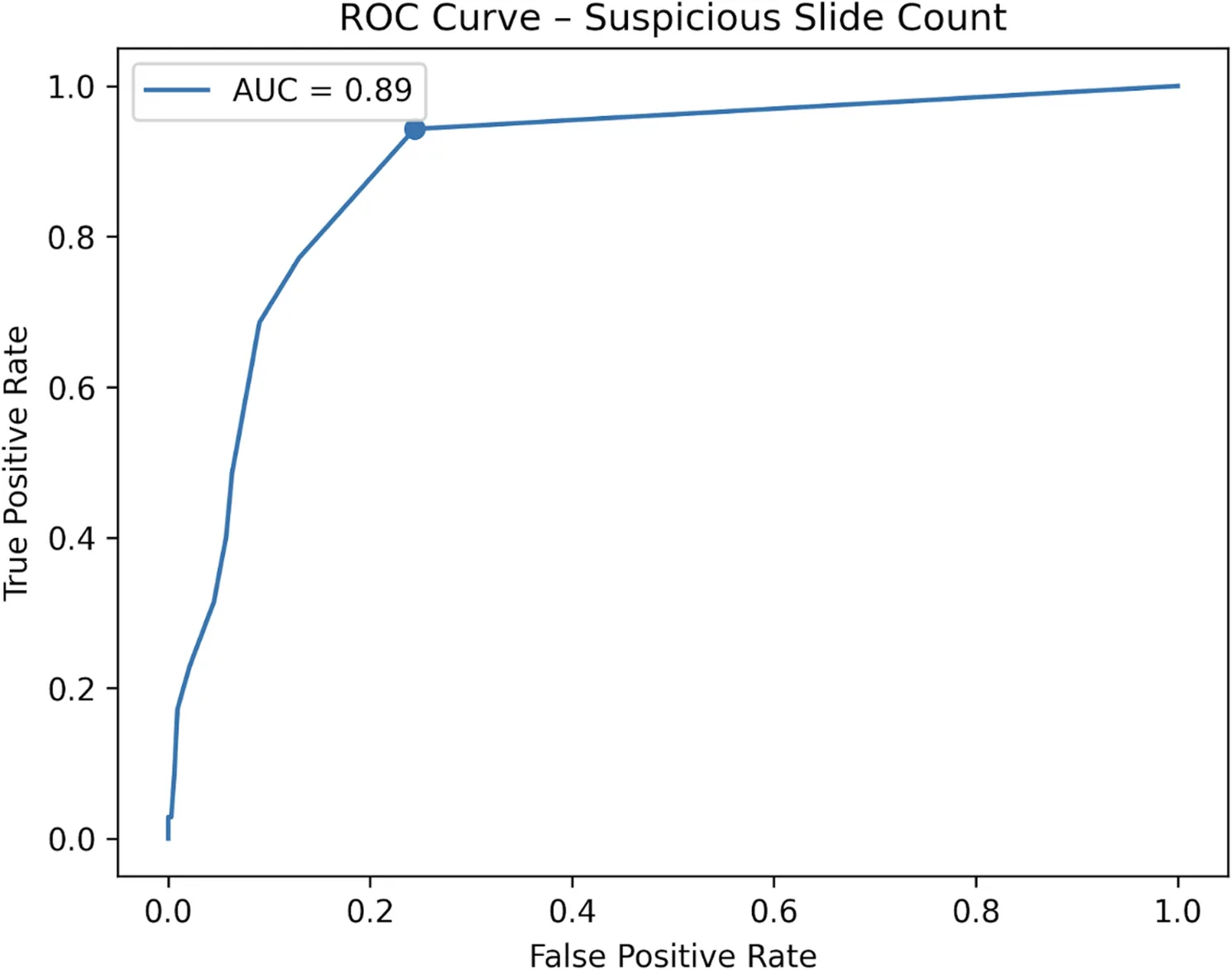
ROC curve for the “Suspicious Slide Count” classification model, showing strong discriminative performance with an AUC of 0.89

The likelihood of malignancy increased progressively with the number of AI-flagged suspicious slides within a case. Cases without suspicious slides demonstrated a malignancy rate of only 0.8%, whereas malignancy rates increased to 13.6% in cases with one suspicious slide, 18.8% in cases with two suspicious slides, and 44.4% in cases with three or more suspicious slides. This stepwise increase demonstrated a statistically significant positive trend according to the Cochran–Armitage trend test (*p* < 0.001), indicating that higher numbers of algorithm-flagged slides were strongly associated with an increased likelihood of malignancy (Fig. 5).

### Analysis of false-positive and false-negative cases

False-positive cases were more frequently observed in areas showing crowded benign glands, basal cell hyperplasia, adenosis-like proliferations, cautery artifact, stromal fragmentation, and inflamed or distorted glandular structures. These findings were particularly common in HoLEP specimens, where laser-related tissue alterations occasionally generated heatmap regions interpreted as suspicious by the algorithm.

False-negative cases were limited to two low-volume HoLEP adenocarcinomas with limited tumor extent and low-grade morphology, suggesting that minimal tumor burden remains a potential limitation for AI-based detection systems trained primarily on biopsy material.

## Discussion

AI-assisted diagnostic systems are increasingly being incorporated into digital pathology workflows, particularly in prostate pathology [13]. Most currently available AI algorithms for prostate cancer detection have been specifically developed and trained using TRUS prostate needle biopsy specimens, where tissue architecture is relatively standardized and well preserved [14, 15]. Several studies have demonstrated excellent diagnostic accuracy of these biopsy-trained algorithms in routine prostate biopsy evaluation, with high sensitivity for adenocarcinoma detection and potential reduction in pathologist workload [11, 12, 15].

In contrast, TURP and HoLEP specimens present unique histomorphological challenges that differ substantially from needle biopsy materials. These specimens are frequently characterized by extensive tissue fragmentation, cautery-related thermal artifact, glandular distortion, stromal disruption, tissue folding, and discontinuous epithelial structures, features that are encountered less commonly and generally to a lesser extent in TRUS biopsy specimens. Such alterations may complicate both human and algorithmic interpretation and therefore represent potential sources of diagnostic error when algorithms are applied outside their original training domain. Consequently, future AI models specifically trained or fine-tuned using TURP and HoLEP datasets incorporating these procedure-related artifacts may achieve improved robustness and diagnostic performance, further enhancing their clinical utility in routine uropathology practice [10, 16, 17].

In the present study, a commercially available AI algorithm originally developed and trained for TRUS-guided prostate biopsy [8] evaluation demonstrated high sensitivity and excellent negative predictive value for the detection of incidental prostatic adenocarcinoma in both TURP and HoLEP materials. The algorithm correctly identified all adenocarcinoma cases in TURP specimens and missed only two low-volume carcinomas in HoLEP specimens. Furthermore, the overall AUC-ROC value of 0.89 indicates strong discriminative capability despite the substantial morphologic complexity of endoscopic prostate resection specimens.

Our findings suggest that biopsy-trained AI algorithms may retain substantial diagnostic utility even when applied to specimen types that differ considerably from their original training datasets. This observation is clinically relevant because incidental adenocarcinoma in TURP and HoLEP materials is often focal, low volume, and identified within large numbers of benign tissue fragments, creating a substantial screening burden in routine uropathology practice. In this context, AI-assisted triage systems may help prioritize suspicious slides for detailed review while reducing unnecessary examination of clearly benign material.

The preserved diagnostic performance observed in this study may reflect the ability of contemporary deep learning models to learn fundamental histomorphologic features of prostatic adenocarcinoma that are not entirely dependent on specimen type. Although the algorithm was originally trained using needle biopsy specimens, many of the cytologic and architectural characteristics of adenocarcinoma remain recognizable in TURP and HoLEP materials despite tissue fragmentation and procedure-related artifacts. Nevertheless, our findings also indicate that specimen-specific fine-tuning or retraining using TURP and HoLEP datasets could further improve specificity, particularly in laser-treated tissues where thermal artifact and architectural distortion contribute to false-positive classifications.

The slightly lower specificity observed in HoLEP specimens compared with TURP specimens likely reflects procedure-specific iatrogenic histomorphologic changes. TURP materials generally consist of relatively intact tissue chips with preserved glandular architecture and recognizable stromal relationships [10]. In contrast, HoLEP specimens frequently demonstrate laser-related thermal artifact, stromal fragmentation, glandular displacement, tissue distortion, and discontinuous epithelial structures. These alterations may obscure benign glandular morphology or simulate infiltrative architectural patterns, thereby increasing false-positive algorithmic interpretations.

Our false-positive analyses further support this interpretation. Suspicious heatmap regions were frequently associated with cautery artifact, crowded benign glands, basal cell hyperplasia, adenosis-like proliferations, stromal fragmentation, and inflamed or distorted glandular structures. These findings suggest that the algorithm may partially misclassify artifact-related architectural irregularities as malignant features, particularly in HoLEP specimens where tissue integrity is more profoundly altered. Such false-positive patterns are consistent with previously reported pitfalls in AI-based prostate pathology systems trained predominantly on conventional biopsy morphology [12, 18].

Despite these specificity limitations, the algorithm demonstrated remarkably high negative predictive values in both specimen groups. This finding is particularly important in TURP and HoLEP practice because these cases often involve numerous slides with relatively low prevalence of malignancy. From a workflow perspective, a high NPV may allow AI-assisted systems to function effectively as screening or triage tools by reliably identifying cases with extremely low probability of carcinoma. Although workflow efficiency and change in turnout time were not directly measured in the present study, the observed diagnostic performance supports the potential clinical utility of such an approach.

Another notable finding was the progressive increase in malignancy rates according to the number of AI-flagged suspicious slides. Cases without suspicious slides demonstrated an extremely low likelihood of carcinoma, whereas cases with three or more suspicious slides showed a markedly increased probability of malignancy. This statistically significant stepwise relationship suggests that AI-generated slide-level outputs may provide not only binary classification but also clinically meaningful risk stratification. Such quantitative slide-level assessment may further improve prioritization strategies in high-volume pathology workflows.

The two false-negative cases were both low-volume Grade Group 1 adenocarcinomas with limited tumor extent and minimal cytologic atypia. Both tumors involved only small portions of the tissue and demonstrated relatively subtle morphologic features. These findings suggest that very small and morphologically inconspicuous tumors remain challenging for AI systems trained predominantly on TRUS biopsy datasets enriched with more overt malignant morphology. Nevertheless, the rarity of false-negative cases in the present cohort supports the overall robustness of algorithmic sensitivity.

Previous studies evaluating AI applications in prostate pathology have largely focused on TRUS biopsy specimens, prostatectomy material, or experimentally developed deep learning models [12, 15, 16, 19]. Comparatively few studies have specifically investigated AI performance in fragmented endoscopic prostate resection specimens, particularly HoLEP materials. Tsuneki et al. demonstrated promising adenocarcinoma classification performance in TURP whole-slide images using transfer learning–based models [6]. However, unlike prior development-focused studies, the present study specifically evaluated the real-world generalizability of a commercially available TRUS biopsy-trained algorithm across both TURP and HoLEP specimens under routine pathology conditions.

An important implication of the present findings is the potential generalizability of biopsy-trained AI systems across different prostate specimen types. Domain shift remains a major challenge in computational pathology, as variations in tissue preparation, specimen architecture, and procedure-related artifacts may substantially affect algorithm performance. Our results suggest that robust AI models may tolerate a considerable degree of domain shift while maintaining clinically useful sensitivity. Nevertheless, external multicenter validation across different scanners, staining protocols, and laboratory workflows will be essential before widespread clinical implementation.

This study has several limitations. First, it was conducted retrospectively at a single institution, which may limit generalizability. Second, the relatively limited number of carcinoma-positive cases restricted detailed subgroup analyses. Third, the evaluated algorithm was originally trained using TRUS-guided biopsy material rather than TURP or HoLEP specimens, which may partly explain the reduced specificity observed in artifact-rich tissues. In addition, the study evaluated standalone algorithmic performance without assessing pathologist–AI interaction or workflow efficiency metrics in real-time diagnostic practice.

Despite these limitations, the study also has important strengths, including the large number of whole-slide images analyzed, inclusion of both TURP and HoLEP specimens, detailed evaluation of false-positive and false-negative cases, and assessment under real-world routine pathology conditions. To our knowledge, relatively few studies have specifically investigated the application of TRUS biopsy-trained AI algorithms in fragmented endoscopic prostate resection materials, particularly HoLEP specimens.

Overall, our findings suggest that AI algorithms originally developed for TRUS-guided prostate biopsy evaluation may also serve as effective clinical support tools for detecting incidental prostatic adenocarcinoma in TURP and HoLEP materials. Integration of such systems into routine digital pathology workflows may improve screening efficiency and reduce diagnostic workload while maintaining high diagnostic safety. Prospective multicenter studies and workflow-based validation analyses will be important for optimizing AI performance specifically for endoscopic prostate resection morphology and for defining the practical role of pathologist–AI collaborative diagnosis in routine uropathology practice.

## Conclusion

A biopsy-trained AI algorithm demonstrated high sensitivity and excellent negative predictive value for detecting incidental prostatic adenocarcinoma in TURP and HoLEP specimens, despite the architectural complexity and procedure-related artifacts of these materials. These findings suggest that such algorithms can be generalized beyond needle biopsy specimens and may serve as effective screening and workflow-assistance tools in routine digital uropathology. Further prospective multicenter studies are needed to validate these results and to evaluate the impact of AI-assisted workflows in clinical practice.

## Data Availability

The datasets generated and/or analyzed during the current study are available from the corresponding author on reasonable request.
